# Comparing prevalence of Iron Deficiency Anemia and Beta Thalassemia Trait in microcytic and non-microcytic blood donors: suggested algorithm for donor screening

**DOI:** 10.4103/0973-6247.53883

**Published:** 2009-07

**Authors:** Aseem K. Tiwari, Iva Chandola

**Affiliations:** *Director-Technical, IMA Blood Bank of Uttarakhand*; 1 *Senior Medical Officer, IMA Blood Bank of Uttarakhand*

**Keywords:** Algorithm, Beta-Thalassemia trait, Ethylenediaminetetraacetic acid, Hemoglobin (Hb), High Performance Liquid Chromatography, Iron Deficiency Anemia, Plasma Ferritin

## Abstract

**Background:**

The prevalence of microcytosis in donors and Iron Deficiency Anemia (IDA) and Beta-Thalassemia trait (BTT) in microcytic and non-microcytic donors has not been studied in India. The present study aims at finding the same.

**Materials and Methods:**

Initially 925 donor samples were evaluated on cell-counter. Of these, 50 were found to be microcytic. These were subjected to Ferritin and HbA2 determination. Subsequently, an additional 51, age-and-sex matched non-microcytic donor samples were selected to serve as controls. These were subjected to the same tests.

**Results:**

The prevalence of microcytosis was 5.4% (50/925). Among the microcytic donors, 52% were IDA, 36% BTT, 8% both, and 4% none. In case of non-microcytic donors 29.4% were IDA, 3.9% BTT, and 66.7% none.

**Conclusions:**

The study revealed a high prevalence of IDA and BTT in blood donors and a higher probability of finding these in the microcytic samples. This prompted authors to suggest an algorithm for screening of blood donors for IDA and BTT. The algorithm recommends doing an hemogram on all donor samples, routinely. Ferritin could be done only in microcytic samples. At levels lower than15 ng/ml, it is diagnosed as IDA, and therefore, HPLC is performed only for non-IDA samples with Ferritin levels higher than 15 ng/ml. By employing this algorithm, a substantial number of IDA and BTT could be diagnosed while keeping the number of Ferritin tests small and the number of HPLC tests even smaller and thus making it cost efficient.

## Introduction

The two most common causes of microcytic anemia are iron deficiency anemia and beta-thalassemia trait.[1‐4] A traditional approach followed by most general practitioners and blood bank physicians is a trial of iron treatment whenever anemia and / or microcytosis are encountered.[5] However populations where Thalassemias are common, like in India, this approach leads to unnecessary iron therapy / iron overload[6‐8] and failure to provide genetic diagnosis / counseling in subjects with BTT. Therefore it is important to diagnose these two most common causes of microcytosis in blood donors.

Limited data was available on prevalence of microcytosis and its causes in blood donors. Therefore in this study microcytosis was evaluated in blood donors, defined as MCV less than 80 fl. The study hypothesized that there is a higher probability of finding IDA and BTT in microcytic samples. Hence Plasma Ferritin and HbA2 determination was done on all microcytic samples. These tests were also done on non-microcytic control samples for comparing prevalence of IDA and BTT in these two groups.

## Materials and Methods

Donor Selection: All accepted and deferred (due to low hemoglobin) voluntary donors visiting at a stand alone blood bank (either in-house or at the outdoor blood drives) were included in the study. The donors were selected randomly during the period between September 2007 and July 2008. The cut off hemoglobin for accepting or deferring a donor was 12.5 gm% as per national guidelines.[9] Hemoglobin (Hb) was measured using the finger-prick method with Hemocue Hb 201+ (HemoCue AB, Angelholm, Sweden) at the time of donation.

Sample Size and Collection: The sample size was determined based on the Krejcie and Morgan[10] criteria, for determining the sample size at 95 and 99% confidence levels. In last few years, every year around ten thousand blood donors between 18 and 60 years have been volunteering at the blood bank for blood donation. Krejcie and Morgan in their criteria of sample selection have suggested a minimum sample of 370 against 10,000 population at 95% confidence level (p < 0.05) and 4950 at 99% confidence level (p < 0.01) for the same target population. Initially 925 test samples were investigated.

Routinely two samples were collected from all blood donors in vacuumized tubes after 350 ml of whole blood donation — one clotted sample for the Transfusion Microbiology Laboratory (TML) tests and another sample in an EDTA tube for the Immunohematology Laboratory (IH). The IH laboratory (EDTA) tube was used for this study. In case of deferred donors the EDTA sample was drawn after obtaining the donors’ oral consent. Since recalling the donor for a second clotted blood sample was difficult and costly, plasma ferritin assay and HPLC were done on the original blood sample when microcytosis was detected in either the accepted or deferred donor.

Hemogram: A complete hemogram was obtained for all samples using SYSMEX KX21 counter (Sysmex Corporation, Kobe, Japan). Diagnosis of microcytosis was made by measuring MCV (less than 80 fl).[11,12] The electronic cell counter was calibrated twice during the course of study. Samples were run on an electronic counter either on the day of collection (day 0) or the day after (day 1) to avoid any changes in MCV that may occur on keeping the samples for long in EDTA. The degree of change in MCV observed from day 2 onward may be considered less than desirable, particularly when the results are borderline normal or abnormal.[13]

Determination of Plasma Ferritin and Hemoglobin Types: Plasma ferritin values were obtained using the chemiluminence method on automated equipment (Vitros Immunodiagnostics, Ortho Clinical Diagnostics, USA). The assays were validated using appropriate controls and calibrators for each lot used. According to the manufacturer’s instructions, the plasma samples showed 15% negative bias for ferritin when compared to the matched serum samples. Therefore the plasma ferritin values were multiplied by a factor of 1.15 to remove this bias. HbA2 quantitation was done by HPLC on an automated system (Bio Rad Variant II, D-10, Bio-Rad Laboratories, CA). Diagnosis of IDA was made based on plasma ferritin values lower than 15 ng ml^-1^. Diagnosis of BTT was made based on HbA2 levels more than 3.5%.

Non-microcytic Controls:_ Subsequently, to carry out a comparative analysis with the microcytic blood donors, an additional 51 age-and-sex matched non-microcytic blood donors were selected. These samples were also subjected to hemogram, plasma ferritin, and HbA2 determination.

Statistical Evaluation: The prevalence data of IDA and BTT in microcytic and non-microcytic samples were compared using the Chi square test. The data was analyzed using SPSS 12.0.

Ethical Issues: The results of a complete hemogram, plasma ferritin, and HPLC were communicated to all blood donors for whom these results were abnormal and all deferred donors from whom samples were obtained. Donors diagnosed with IDA or BTT were recalled and counseled. The ethical clearance for this study was obtained from the Institutional Ethical Committee. There was no potential conflict of interest relevant to this study.

## Results

In the initial 925 donor samples evaluated, there were 688 males (74.4%) and 237 (25.6%) females. Seven hundred and sixty-three were accepted donors and 162 were deferred donors. The mean age of the donors was 28.9 years. The data on first-time and repeat donors was not tabulated / evaluated. Out of these 925 samples 50 were found to be microcytic (< 80 fL). Therefore, the prevalence of microcytosis in donors worked out to be 5.4%.

As Table 1 depicts, out of the 50 microcytic donors, 52% were diagnosed with IDA, 36% with BTT, 8% showed both IDA and BTT, and 4% did not show either of these.

In the 51 age-and-sex matched non-microcytic donors, 29.4% had IDA, 3.9% had BTT, and 66.7% had none.

**Table 1:** Prevalence of IDA and BTT in blood donors

Type of donors	Number	IDA	BTT	Both	None
Microcytic	50	26 (52%)	18 (36%)	4 (8%)	2 (4%)
Non-microcytic	51	15 (29.4%)	2 (3.9%)	0 (0%)	34 (66.7%)

The prevalence of both IDA and BTT in the microcytic samples was significantly higher when compared with the non-microcytic samples (P = 0.000).

## Discussion

The study investigates microcytosis in blood donors. Microcytosis is the most common red cell change observed during a routine blood count and is an important indicator of anemia. Anemia, to date, remains the most important cause of deferral in blood donors, and therefore, investigating microcytosis in donors, not only helps in identifying the cause of anemia but also provides right treatment and counseling to the donor. This goes a long way in maintaining a dedicated pool of donors.

In several studies, red blood cells are described as being microcytic when the mean corpuscular volume is less than 80 fl.[11,12,14,15] MCV measurement by cell counter is direct, rapid, inexpensive, and automated. The prevalence of microcytosis in donors in this study was 5.4%. This was slightly lower than that found in the high school students of Hong Kong (8.3%), in a study by Yu-Lung Lau *et al*. (1997), who also followed the same criteria for MCV (< 80 fl).[16] This difference could be because of the different sets of population and different mean age of subjects in this study.

Plasma ferritin has been used to diagnose IDA because the ferritin level is considered to be the single, most powerful test for its diagnosis[17‐19] Ferritin is independent of external contamination of blood samples, diurnal variation, and concurrent iron therapy. Even though plasma ferritin is an acute phase reactant that can be elevated in various inflammatory conditions, as this study group comprised of healthy donor population, the probability of inflammation was negligible. Chemiluminescence for Ferritin assay was preferred over other methods because of its simplicity and sensitivity.[20] For diagnosis of IDA, plasma ferritin threshold of 15 ng ml^-1^ was used in this study, as suggested by Susan F Clark (2008).[21]

High Performance Liquid Chromatography was used for quantization of HbA2 because of the simplicity of sample preparation, superior resolution, and accuracy, combined with complete automation of the method.[22] Diagnosis of BTT was based on levels of HbA2 greater than 3.5 %.[23] Reduction of HbA2 because of coincident iron deficiency did not preclude detection of BTT.[24]

**Figure 1:**
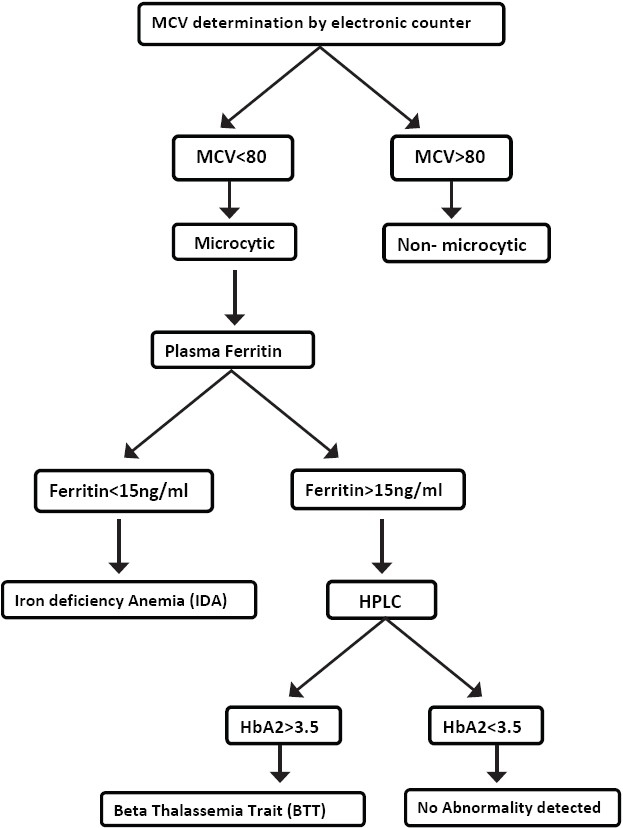
Algorithm for mass screening of blood donors for IDA and BTT

The present study illustrates two important aspects; one, the prevalence of IDA and BTT was high among blood donors and second, the probability of both IDA and BTT in microcytic samples was significantly higher as compared to non-microcytic samples. Several studies including two recent Indian studies[25,26] emphasized the high prevalence of IDA in blood donors. The prevalence of BTT in blood donors in India is being reported for the first time here in this study. Based on these findings, the authors have suggested an algorithm as shown in figure 1. The algorithm recommends conducting a hemogram on all donor samples, routinely. Plasma ferritin could be done only in microcytic samples. Those with ferritin levels less than 15 ng/ml are diagnosed as IDA. HPLC is performed only for non-IDA samples, with ferritin levels higher than 15 ng/ml, as BTT is more likely in samples with higher ferritin levels. The same recommendation has been put forward by Loria (1978) and Hershko (1979).[27,28]

The authors did not find any algorithm for diagnosing IDA and BTT in blood donors in India. However, there were two published algorithms from other countries in context to IDA and BTT. A Canadian study by Kiss *et al*. (2000)[15] has also used 80 fl as the initial screening tool in patients. Their algorithm demonstrates that a high index of suspicion based on ethnic background and low MCV is a relevant approach to the effective investigation of the thalassemia trait. The present study done on blood donors suggests an algorithm that increases the probability of finding both BTT and IDA. However, this study does not consider the ethnic background of donors. An American study by Pearson *et al*. (1973)[11] used 79 fl as the initial screening tool. Their algorithm suggests doing HbA2 electrophoresis on all microcytic samples and serum iron for all non-BTT samples. The algorithm in the present study recommends doing plasma ferritin and HPLC for IDA and BTT diagnosis, respectively, which are newer and more accurate tests available, as compared to serum iron and electrophoresis used by Pearson *et al*. The order of the tests performed has been reversed in the present study; HPLC is performed only for non-IDA samples, with ferritin levels higher than 15 ng/ml, as BTT is more likely in samples with higher ferritin levels.

By employing this algorithm, a substantial number of IDA and BTT could be diagnosed while keeping the number of Ferritin tests small and the number of HPLC tests even smaller, thus making it cost efficient.
